# Comparison of Immune Responses and Safety Profiles Following a Fourth Heterologous Dose (Second Booster) with mRNA-1273 in Individuals Previously Vaccinated with Two Doses of CoronaVac and a Booster Dose of Either AZD1222 or BNT162b2

**DOI:** 10.3390/vaccines14040348

**Published:** 2026-04-15

**Authors:** Auchara Tangsathapornpong, Sira Nanthapisal, Waraphon Fukpho, Pornumpa Bunjoungmanee, Yamonbhorn Neamkul, Kanassanan Pontan, Arthit Boonyarangkul, Supattra Wanpen, Kanokporn Thongphubeth, Phuntila Tharabenjasin, Peera Jaru-Ampornpan

**Affiliations:** 1 Department of Pediatrics, Faculty of Medicine, Thammasat University, Khlong Nueang, Khlong Luang, Pathumthani 12120, Thailand; 2 Research Unit in Infectious and Immunology, Faculty of Medicine, Thammasat University, Khlong Nueang, Khlong Luang, Pathumthani 12120, Thailand; 3 Clinical Research Center, Faculty of Medicine, Thammasat University, Khlong Nueang, Khlong Luang, Pathumthani 12120, Thailand; 4 Thammasat University Hospital, Thammasat University, Khlong Nueang, Khlong Luang, Pathumthani 12120, Thailand; 5 Department of Obstetrics and Gynecology, Faculty of Medicine, Thammasat University, Khlong Nueang, Khlong Luang, Pathumthani 12120, Thailand; 6 Chulabhorn International College of Medicine, Thammasat University, Khlong Nueang, Khlong Luang, Pathumthani 12120, Thailand; 7 Virology and Cell Technology Research Team, National Center for Genetic Engineering and Biotechnology (BIOTEC), Khlong Nueang, Khlong Luang, Pathumthani 12120, Thailand

**Keywords:** SARS-CoV-2 vaccine, CoronaVac, AZD1222, BNT162b2, mRNA-1273, booster dose

## Abstract

***Background/Objectives***: Our previous study demonstrated that while the third SARS-CoV-2 booster effectively enhanced immunity against the Delta subvariant, its protection declined over time. This study aimed to evaluate and compare the humoral and cellular immune responses, as well as reactogenicity, of the mRNA-1273 vaccine administered as a fourth booster in healthy Thai adults previously vaccinated with two doses of CoronaVac (CV) followed by a third dose of either AZD1222 (AZ) or BNT162b2 (BNT). ***Methods***: Participants received a single 100 µg (0.5 mL) intramuscular dose of mRNA-1273. Blood samples were collected at baseline (D0), D14, D90, and D180 to assess anti-RBD IgG, conduct a surrogate virus neutralization test (sVNT) against the Delta and Omicron variants, and assess IFN-γ levels and reactogenicity. ***Results***: Both 2CV/AZ- and 2CV/BNT-primed groups exhibited comparable local and systemic reactogenicity. The fourth mRNA-1273 dose markedly increased Delta variant inhibition within 14 days in both groups and remained at high levels at Days 90 and 180. sVNT inhibition against Omicron rose similarly in both groups at Day 14; it declined sharply by Days 90 and 180, with the 2CV/AZ-primed group showing significantly lower levels than the 2CV/BNT-primed group. Baseline anti-RBD IgG levels were lower in the 2CV/AZ group (*p* = 0.003) but surpassed those of the 2CV/BNT group by Day 14, with no significant differences at later time points. IFN-γ responses followed a similar pattern to anti-RBD IgG ***Conclusions***: A heterologous fourth mRNA-1273 booster in both 2CV/AZ- and 2CV/BNT-primed groups effectively enhances B-cell and T-cell responses against SARS-CoV-2. However, emerging variants such as Omicron may still pose challenges. The trial was registered with the Thai Clinical Trials Registry: the name of the registry: “The comparison of immune response to the 4th dose booster with mRNA-1273 COVID-19 vaccine in individuals who had received 2 doses of CoronaVac and booster with ChAdOx-1 or BNT162b2 COVID-19 vaccine”, TCTR20220205002 on 5 February 2022.

## 1. Introduction

Coronavirus disease 2019 (COVID-19) is caused by infection with the severe acute respiratory syndrome coronavirus 2 (SARS-CoV-2), which was first identified in Wuhan, the capital of Hubei Province, People’s Republic of China [1]. The novel coronavirus rapidly spread throughout China and subsequently across the globe, including Thailand, where the first confirmed case was reported in early January 2020 [2]. Thailand experienced a significant outbreak in 2021. During that year, the emergence of new SARS-CoV-2 variants, specifically the Delta and Omicron subvariants, was first detected in May and November, respectively. These variants have been major contributors to successive waves of infection in Thailand and other countries [3,4].

Vaccination against SARS-CoV-2 has been recognized as one of the most effective strategies to mitigate the spread of COVID-19. Currently, a variety of COVID-19 vaccines are available globally [5]. Thailand’s national COVID-19 vaccination campaign began in March 2021. The first vaccine administered was an inactivated SARS-CoV-2 vaccine (CoronaVac, Sinovac Life Sciences, Beijing, China), delivered in a two-dose regimen spaced 21 to 28 days apart. Additionally, a two-dose schedule of the adenoviral vector vaccine ChAdOx1 nCoV-19 (AZD1222, Oxford/AstraZeneca) was widely implemented during the early phase of the pandemic. Later in 2021, Thailand expanded its vaccine portfolio to include the nucleoside-modified mRNA vaccine BNT162b2 (BioNTech/Pfizer), the inactivated BBIBP-CorV vaccine (Sinopharm), and the mRNA-1273 vaccine (Moderna) [6,7].

As the protective efficacy of COVID-19 vaccines against severe disease gradually diminishes over time, remaining at approximately ≥70% for up to six months, booster doses following the primary vaccination series are thus recommended [8,9]. A systematic review indicated that the decline in vaccine effectiveness is attributable to genuine waning immunity, characterized by a reduction in vaccine-induced immunological protection, and/or to the increasing prevalence of highly transmissible variants such as Delta and Omicron [4,8,10], which are mutations of the virus that enhance viral transmissibility compared to earlier strains of SARS-CoV-2. The Omicron variant of SARS-CoV-2 has been reported to be approximately twice as transmissible as the original strain, with enhanced adaptability to human hosts, an increased risk of reinfection, and reduced vaccine-induced protection [11,12,13].

In terms of vaccination strategies, previous studies have demonstrated that heterologous booster regimens elicit more robust immune responses compared to homologous boosters, thereby offering enhanced protection against SARS-CoV-2 [14]. Administration of a third dose using either AZD1222 (AstraZeneca (Cambridge, UK)) or the BNT162b2 mRNA vaccine (Comirnaty, Pfizer-BioNTech, United States) has been shown to significantly augment immunogenicity and expand both B cell and T cell responses against circulating variants of concern [15,16,17,18]. Nevertheless, high susceptibility to SARS-CoV-2 infection has been observed in the Thai population, particularly among healthcare workers, who initially received two doses of the inactivated vaccine CoronaVac followed by a heterologous booster with AZD1222 or BNT162b2.

To mitigate the risk of another surge in COVID-19-related morbidity and mortality, as well as to prevent recurrent outbreaks, the U.S. Centers for Disease Control and Prevention (CDC) recommended administration of a fourth vaccine dose (or a first booster dose for individuals who had received a three-dose primary series) in October 2021 [19,20]. With this guidance, the Thai Ministry of Public Health initiated the rollout of a fourth (second booster) dose, prioritizing residents of long-term care facilities who had previously completed a three-dose regimen. As of 1 November 2021, Moderna’s COVID-19 vaccine, an alternative to the Sinovac, AstraZeneca, and Pfizer vaccines, has been available in Thailand. Moreover, our research group recently provided evidence demonstrating the high immunogenicity of the mRNA-1273 booster following two doses of either CoronaVac or AZD1222 in the Thai population [21]. However, we observed a significant decline in the immunological effectiveness of the third booster dose over time, as measured by anti-receptor-binding domain (RBD) IgG antibody levels, surrogate virus neutralization tests, and interferon-gamma (IFN-γ) responses at days 14 and 90 post-vaccination, respectively [21]. These findings underscore the necessity of strategies aimed at prolonging immune protection against SARS-CoV-2 to ensure sustained defense against COVID-19.

As to now, clinical trials evaluating the immunogenicity and safety of a heterologous fourth booster dose with mRNA-1273 among individuals in the Thai population who previously received two doses of CoronaVac followed by a third booster with either AZD1222 or BNT162b2 remain lacking. Therefore, the present study aimed to compare the humoral and cellular immune responses, as well as the reactogenicity, of the mRNA-1273 COVID-19 vaccine administered as a fourth-dose booster in healthy Thai adults who had previously received two doses of CoronaVac followed by a third dose of either AZD1222 or BNT162b2. We hypothesized that the heterologous mRNA-1273 booster would enhance immune protection against both the Delta and Omicron variants of SARS-CoV-2 across both vaccination regimens.

## 2. Materials and Methods

### 2.1. Ethics Approval, Study Design, and Participants

This prospective observational study was conducted at Thammasat University Hospital between February and July 2022. All study activities and procedures were carried out in accordance with the ethical guidelines of the Human Research Ethics Committee, Faculty of Medicine, Thammasat University (Institutional Review Board approval no. 019/2565) and the rules of the Declaration of Helsinki in revised version 2013. The trial was registered with the Thai Clinical Trials Registry (The name of the registry: “The comparison of Immune response to the 4th dose booster with mRNA-1273 COVID-19 vaccine in individuals who had received 2 doses of CoronaVac and booster with ChAdOx-1 or BNT162b2 COVID-19 vaccine” with registration number TCTR20220205002 on 5 February 2022). Prior to participation, all individuals were fully informed about the study protocol and potential adverse effects, and written informed consent was obtained from each participant before any study-related procedures were undertaken.

A total of 100 adults aged 18 years and older were enrolled, all of whom had previously received two doses of CoronaVac followed by a third booster dose administered more than 90 days prior, with either the AZD1222 vaccine (2CV/AZ; *n* = 50) or the BNT162b2 vaccine (2CV/BNT; *n* = 50). Exclusion criteria included: (1) a history of COVID-19 infection or receipt of any vaccine within 14 days prior to enrollment; (2) prior receipt of more than three doses of any COVID-19 vaccine; (3) current use of immunosuppressive therapy or systemic corticosteroids; (4) presence of acute illness or a body temperature ≥ 38 °C on the day of vaccination; and (5) pregnancy with a gestational age of less than 12 weeks.

All participants received a single 100 μg dose of the mRNA-1273 vaccine as a fourth-dose booster from a licensed nurse/physician. Blood samples were collected at four times to assess immune responses: baseline (Day 0; D0) and at 14, 90, and 180 days post-vaccination. To exclude asymptomatic SARS-CoV-2 infection, serum IgG against the nucleocapsid (N) protein was evaluated at baseline.

### 2.2. Study Procedures

Demographic and baseline characteristics, including age, sex, height, weight, and relevant clinical data such as comorbidities, were assessed to ensure compliance with inclusion and exclusion criteria. Participants received a 100 μg single intramuscular dose (0.5 mL) of the mRNA-1273 COVID-19 vaccine, manufactured by Moderna [22]. (Lot numbers: 30125BA and FH6387), administered in the deltoid muscle. Following vaccination, all participants were monitored for a minimum of 30 min to observe for any immediate adverse reactions. Diary cards were provided for participants to record local and systemic post-vaccination symptoms, including pain, redness, or swelling at the injection site, as well as fever, headache, myalgia, arthralgia, fatigue, diarrhea, and vomiting for up to 7 days following immunization.

**Figure 1:**
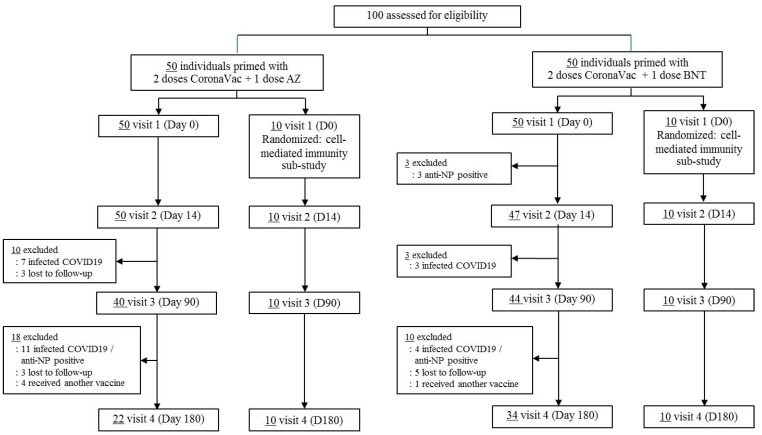
Consolidated Standards of Reporting Trials diagram.

To assess the immune response to the booster vaccination, blood samples were collected from all participants at four time points: Visit 1 (Day 0, pre-vaccination baseline), Day 14 (D14, Visit 2), Day 90 (D90, Visit 3), and Day 180 (D180, Visit 4). The samples were processed on-site for serum separation, after which the serum was transported to designated laboratories for immunogenicity analysis. Immunoglobulin G (IgG) antibodies targeting the receptor-binding domain (RBD) of the S1 subunit of the SARS-CoV-2 spike protein were measured, along with surrogate viral neutralization titers (sVNT) against SARS-CoV-2 variants, B.1.617.2 (Delta) and B.1.1.529 (Omicron), include its subvariants BA.1, BA.2, and BA.4/5. Participants with laboratory evidence of prior SARS-CoV-2 infection, indicated by a positive IgG response to the nucleocapsid (N) protein, were excluded from the analysis. A randomized subset of ten participants from each group was selected for a sub-study to assess T cell-mediated immune responses. Blood samples were collected at baseline (pre-vaccination), as well as on Days 14, 90, and 180 post-vaccination, to evaluate interferon-gamma (IFN-γ) responses using the IFN-γ Release Assay (IGRA) (Figure 1).

### 2.3. Immunogenicity Outcomes

#### 2.3.1. Quantitative Receptor Binding Domain IgG (Anti-RBD IgG) and Anti-Neucleocapsid Protein (Anti-Np)

The humoral immune response to SARS-CoV-2 was assessed by measuring antibodies against the receptor-binding domain (RBD) of the spike protein using the BIOTEC COVID-19 IgG ELISA kit (Abbott Diagnostics, Chicago, IL, USA). Results were reported in binding antibody units per milliliter (BAU/mL). Samples with anti-RBD IgG levels exceeding 40,000 AU/mL were diluted appropriately according to the manufacturer’s instructions to fall within the assay’s quantifiable range. To detect prior or asymptomatic SARS-CoV-2 infection, antibodies against the nucleocapsid protein (anti-N) were quantified using the Elecsys Anti-SARS-CoV-2 assay (Roche, Basel, Switzerland), an electrochemiluminescence immunoassay (ECLIA) that detects total antibodies, including IgA, IgG, and IgM. A value greater than 10 AU/mL was considered positive.

#### 2.3.2. Surrogate Virus Neutralization Test (sVNT)

Neutralizing antibodies against SARS-CoV-2 were assessed using a surrogate virus neutralization test (sVNT) to detect antibodies specific to the Delta and Omicron variants. The sVNT is an ELISA-based assay that measures the ability of neutralizing antibodies to block the interaction between the receptor-binding domain (RBD) of the SARS-CoV-2 spike protein and the human angiotensin-converting enzyme 2 (hACE2) receptor. This assay was performed at the National Center for Genetic Engineering and Biotechnology (BIOTEC), Thailand. At the time of testing, purified recombinant hACE2 and the RBD of the SARS-CoV-2 Delta and Omicron variant spike protein were used in the assay.

Diluted serum samples were mixed with horseradish peroxidase (HRP)-conjugated receptor-binding domain (RBD) protein. The mixture was incubated and then added to 96-well plates pre-coated with 0.1 µg of recombinant human ACE2 (hACE2) ectodomain per well (GenScript). The level of neutralizing antibodies was quantified and expressed as percent signal inhibition (% inhibition) using the following equation:

$$\%inhibition=100\times \left[1-\frac{sampleOD450}{negativeOD450}\right]$$

A surrogate virus neutralization test (sVNT) percent inhibition of ≥80% for the Delta variant and ≥50% for the Omicron variant was defined as the threshold for a positive neutralizing antibody response.

#### 2.3.3. Interferon-Gamma (IFN-γ) Release Assay (IGRA) to Evaluate T Cell Responses

SARS-CoV-2-reactive T-cell responses were assessed using the Euroimmun^®^ SARS-CoV-2 IGRA kit (Euroimmun, Lübeck, Germany). This assay evaluates cell-mediated immunity by measuring IFN-γ released from SARS-CoV-2-specific memory T cells after stimulation with viral antigens. Fresh heparinized whole blood was stimulated with SARS-CoV-2 antigens, and IFN-γ released by antigen-specific memory T cells in plasma supernatant was quantified by ELISA using the Euroimmun Analyzer platform according to the manufacturer’s instructions. Results were interpreted as follows: an IFN-γ response (stimulated minus blank) of <100 mIU/mL was considered negative, 100–200 mIU/mL as borderline, and >200 mIU/mL as positive.

### 2.4. Reactogenicity

Solicited local and systemic adverse reactions were assessed from Day 0 to Day 7 post-vaccination using a self-reported diary card. The severity of reactions was graded according to the Guidance for Industry: Toxicity Grading Scale for Healthy Adult and Adolescent Volunteers Enrolled in Preventive Vaccine Clinical Trials [23]. A grading scale was applied as follows: grade 0 indicated the absence of symptoms; grade 1 denoted mild symptoms that did not interfere with daily activities, or included vomiting 1–2 times per day or diarrhea occurring 2–3 times per day; grade 2 reflected moderate symptoms that interfered with activities or required symptomatic treatment, or involved vomiting more than twice per day or diarrhea 4–5 times per day; grade 3 was assigned to severe symptoms that were incapacitating or to diarrhea exceeding five episodes per day; and grade 4 represented potentially life-threatening events necessitating hospitalization or emergency medical intervention. Fever was graded separately as follows: grade 1 (38.0–38.4 °C); grade 2 (38.5–38.9 °C); grade 3 (39.0–40.0 °C); and grade 4 (greater than 40.0 °C). Unsolicited adverse events were also recorded by the study investigators during each scheduled visit.

### 2.5. Statistical Analysis

Demographic characteristics, laboratory parameters, and other continuous variables were summarized as medians with interquartile ranges (IQR), while categorical variables were presented as frequencies and percentages.

Differences between groups were assessed using the Wilcoxon rank-sum test for non-normally distributed continuous variables, including age, body mass index (BMI), and the interval between the third dose of AZD1222 or BNT162b2 and the mRNA1273 booster. Categorical variables, including sex, BMI categories, comorbidities, and adverse events, were compared using the chi-square (χ^2^) test or Fisher’s exact test, as appropriate based on sample size.

Antibody titers were log-transformed prior to analysis, and results were expressed as geometric means with two-sided 95% confidence intervals (CIs), calculated by back-transformation (antilogarithm) of the mean differences obtained from independent two-sample *t*-tests.

All *p*-values were two-sided, and values less than 0.05 were considered statistically significant. Statistical analyses were conducted using GraphPad Prism 9.0 (GraphPad Software Inc., San Diego, CA, USA).

## 3. Results

### 3.1. Baseline Characteristics

Figure 1 presents the Consolidated Standards of Reporting Trials (CONSORT) diagram for this study. A total of 100 adults were initially enrolled, consisting of 50 individuals who had previously received two doses of CoronaVac followed by a third booster dose with AZD1222 (2CV/AZ), and 50 individuals who had received two doses of CoronaVac followed by a booster dose of BNT162b2 (2CV/BNT). Three participants in the 2CV/BNT group were excluded due to positive anti-nucleocapsid (anti-Np) antibodies at baseline. Between D14 and D90, 10 participants reported COVID-19 infection (7 from the 2CV/AZ group and 3 from the 2CV/BNT group). Additionally, 3 participants from the 2CV/AZ group were lost to follow-up during this period. From D90 to D180, 15 participants (11 in the 2CV/AZ group and 4 in the 2CV/BNT group) were diagnosed with COVID-19 or tested positive for anti-Np. Eight participants were lost to follow-up during this interval (3 from the 2CV/AZ group and 5 from the 2CV/BNT group), and 5 participants received an additional COVID-19 booster vaccine before D180 (4 from the 2CV/AZ group and 1 from the 2CV/BNT group). As a result, 22 participants in the 2CV/AZ group and 34 in the 2CV/BNT group completed the study.

Table 1 presents the baseline characteristics and demographic data of the participants. The overall median age was 39 years (interquartile range [IQR]: 31–44), with a median of 40 years (IQR: 31–45) in the 2CV/AZ-primed group and 37.5 years (IQR: 29–43) in the 2CV/BNT-primed group. Females comprised the majority of participants (79% overall), with a higher proportion in the 2CV/AZ group (88%) compared to the 2CV/BNT group (74.47%), though this difference was not statistically significant (*p* = 0.118).

The median body mass index (BMI) was 24.03 kg/m^2^ (IQR: 20.57–28.37) in the 2CV/AZ-primed group and 22.53 kg/m^2^ (IQR: 20.06–25.69) in the 2CV/BNT-primed group (*p* = 0.169). Overall, 25 participants (26.32%) had at least one comorbidity, 15 in the 2CV/AZ group and 10 in the 2CV/BNT group (*p* = 0.36). Reported comorbidities included allergic rhinitis (10.53%), diabetes mellitus (5.26%), obesity (4.21%), cardiovascular disease or hypertension (3.16%), thyroid disorders (2.06%), and chronic lung disease (1.05%). The median interval between the third dose (AZD1222) and the mRNA-1273 booster in the 2CV/AZ-primed group was 184 days (IQR: 149–190), which was significantly longer than the interval between BNT162b2 and the mRNA-1273 booster in the 2CV/BNT-primed group, at 162 days (IQR: 158–164) (*p* = 0.005).

### 3.2. Reactogenicity

**Figure 2:**
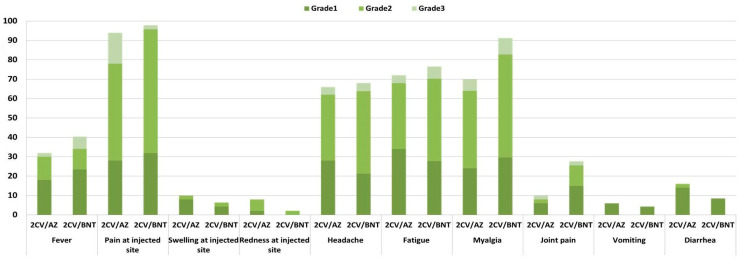
Individuals primed with either 2CoronaVac/AZ (*n* = 47) or 2CoronaVac/BNT (*n* = 50) experienced similar local and systemic reactogenicity within 7 days after receiving a fourth mRNA-1273 booster. Data are presented as percentages in a bar graph.

The number of participants who experienced local and systemic reactogenicities is presented in Figure 2 and Table S1. Adverse events within the first week after receiving the mRNA-1273 booster were recorded using participant diary cards. Most adverse reactions were mild and resolved spontaneously. The most commonly reported reaction was pain at the injection site (95.88%), followed by myalgia (79.38%), fatigue (74.23%), headache (67.01%), fever (36.08%), arthralgia (18.56%), diarrhea (12.37%), swelling at the injection site (8.25%), redness at the injection site (5.15%), and vomiting (5.15%). Severe (Grade 3) adverse events were less frequent and included pain at the injection site (9.28%), myalgia (7.22%), fatigue (5.15%), headache (4.12%), fever (4.12%), and arthralgia (2.06%). Overall, the 2CV/AZ-primed and 2CV/BNT-primed groups experienced similar rates and patterns of local and systemic reactogenicity following the mRNA-1273 booster. Of note, rare adverse events, including autoimmune reactions and thrombotic events, were not observed in our study.

Statistical differences between groups were assessed using the chi-square or Fisher’s exact test, as appropriate. Adverse events were graded according to the U.S. Department of Health and Human Services, Food and Drug Administration (FDA), Center for Biologics Evaluation and Research (CBER) toxicity grading scale for healthy adult and adolescent volunteers in preventive vaccine trials (September 2007): Grade 1 (mild), transient or mild discomfort with no limitation of activity; Grade 2 (moderate), some limitation of activity; and Grade 3 (severe), marked limitation of activity or requiring medical intervention.

### 3.3. Immunogenicity

#### 3.3.1. SARS-CoV-2 Neutralizing Antibody by Surrogate Virus Neutralization Test (sVNT)

**Figure 3:**
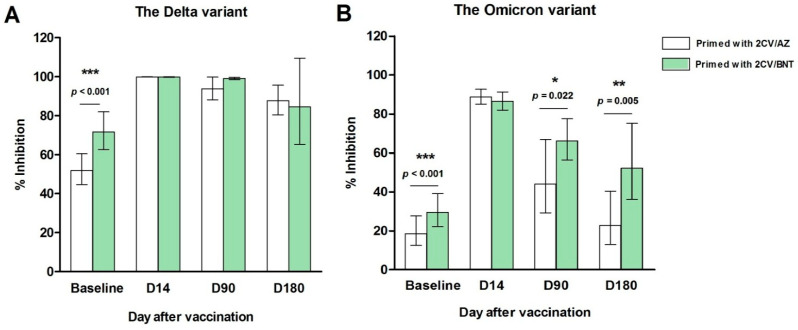
Surrogate virus neutralization test results against the Delta (**A**) and Omicron (**B**) variants before (*n* = 50 per group), and days 14 (D14), D90, and D180 after the fourth booster vaccination with mRNA-1273 in participants primed with either 2 doses of CoronaVac followed by AZ (2CoronaVac/AZ) or 2 doses of CoronaVac followed by BNT (2CoronaVac/BNT). Sample sizes were D14: *n* = 50 (AZ) and 47 (BNT); D90: *n* = 40 (AZ) and 44 (BNT); D180: *n* = 22 (AZ) and 34 (BNT). Data are presented as geometric means with 95% CIs, and mean differences were analyzed using independent *t*-tests. * *p* < 0.05, ** *p* < 0.01, and *** *p* < 0.001 compared with primed with 2CoronaVac/AZ group (white bar).

As shown in Figure 3, the geometric means (GMs) of surrogate virus neutralization test (sVNT) inhibition against the Delta variant significantly increased 14 days after the mRNA-1273 booster in both groups. In the 2CV/AZ-primed group, sVNT rose from 51.93% inhibition (95% CI: 44.63–60.43) at baseline to 99.88% (95% CI: 99.83–99.93) at D14. Similarly, in the 2CV/BNT-primed group, sVNT increased from 71.68% inhibition (95% CI: 62.58–81.98) to 99.79% (95% CI: 99.61–99.96). By D90 and D180, a slight decline in sVNT levels was observed. In the 2CV/AZ group, inhibition decreased to 93.76% (95% CI: 88.10–99.77) and 87.66% (95% CI: 80.38–95.61), respectively. In the 2CV/BNT group, inhibition dropped to 99.10% (95% CI: 98.47–99.73) at D90 and 84.51% (95% CI: 65.22–109.52) at D180. At baseline, the GM of sVNT inhibition in the 2CV/AZ-primed group was significantly lower than that in the 2CV/BNT-primed group (*p* < 0.001). However, from D14 to D180 post-mRNA-1273 booster, sVNT responses were comparable between the two groups.

Regarding the Omicron variant, geometric means (GMs) of sVNT inhibition significantly increased 14 days after the mRNA-1273 booster in both groups. In the 2CV/AZ-primed group, inhibition rose from 18.57% at baseline (95% CI: 12.45–27.69) to 88.78% (95% CI: 85.01–92.72) at D14. Similarly, in the 2CV/BNT-primed group, inhibition increased from 29.56% (95% CI: 22.35–39.10) to 86.50% (95% CI: 81.97–91.28). By D90 and D180, a gradual decline in sVNT responses was observed. In the 2CV/AZ group, inhibition decreased to 44.20% (95% CI: 29.20–66.92) at D90 and further to 22.88% (95% CI: 12.97–40.36) at D180. In the 2CV/BNT group, inhibition declined to 66.15% (95% CI: 56.41–77.57) at D90 and 52.10% (95% CI: 36.11–75.17) at D180. At baseline, the geometric mean (GM) of sVNT inhibition against the Omicron variant was significantly lower in the 2CV/AZ-primed group compared to the 2CV/BNT-primed group (*p* < 0.001). Fourteen days after the mRNA-1273 booster, sVNT levels increased significantly in both groups, with no statistically significant difference observed between them. However, at D90 and D180 post-booster, the GM of sVNT inhibition against Omicron remained significantly lower in the 2CV/AZ group compared to the 2CV/BNT group (*p* = 0.022 and *p* = 0.005, respectively).

#### 3.3.2. Quantitative IgG Against Receptor-Binding Domain (Anti-RBD IgG)

**Figure 4:**
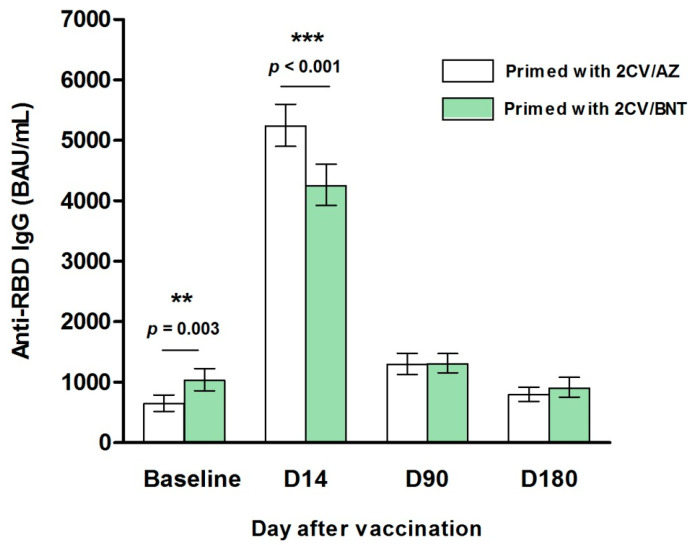
The geometric mean titer of anti-SARS-CoV2-receptor binding domain IgG before, and days 14 (D14), D90, and D180 after the fourth booster vaccination with mRNA-1273 in participants primed with either 2 doses of CoronaVac followed by AZ (2CoronaVac/AZ) or 2 doses of CoronaVac followed by BNT (2CoronaVac/BNT). Sample sizes were D14: *n* = 50 (AZ) and 47 (BNT); D90: *n* = 40 (AZ) and 44 (BNT); D180: *n* = 22 (AZ) and 34 (BNT). Data are presented as geometric means with 95% CIs, and mean differences were analyzed using independent *t*-tests. ** *p* < 0.01 and *** *p* < 0.001 compared with primed with 2CoronaVac/AZ group (white bar).

At baseline, the geometric mean titer (GMT) of anti-RBD IgG was significantly higher in the 2CV/BNT-primed group compared to the 2CV/AZ-primed group (*p* = 0.003). By D14 following the mRNA-1273 booster, anti-RBD IgG levels increased markedly in both groups. In the 2CV/AZ group, GMT rose from 640.70 BAU/mL (95% CI: 518.95–791.02) to 5238.73 BAU/mL (95% CI: 4902.08–5598.51), while in the 2CV/BNT group, it increased from 1025.99 BAU/mL (95% CI: 857.18–1228.04) to 4251.07 BAU/mL (95% CI: 3922.73–4606.90). Interestingly, despite lower baseline levels, the GMT of anti-RBD IgG at D14 post-booster was significantly higher in the 2CV/AZ-primed group compared to the 2CV/BNT-primed group (*p* < 0.001) (Figure 4).

At D90 and 180 following the mRNA-1273 booster, anti-RBD IgG levels declined in both groups. In the 2CV/AZ-primed group, the geometric mean titers (GMTs) decreased to 1291.71 BAU/mL (95% CI: 1129.77–1476.87) at D90 and to 793.88 BAU/mL (95% CI: 683.98–921.44) at D180. Similarly, in the 2CV/BNT-primed group, GMTs declined to 1307.03 BAU/mL (95% CI: 1159.70–1473.07) at D90 and to 903.69 BAU/mL (95% CI: 756.60–1079.38) at D180. The GMTs of anti-RBD IgG at both D90 and D180 were comparable between the two groups, with no significant differences observed (*p* = 0.881 and *p* = 0.199, respectively) (Figure 4).

### 3.4. Cell-Mediated Immune Response by IGRA

**Figure 5:**
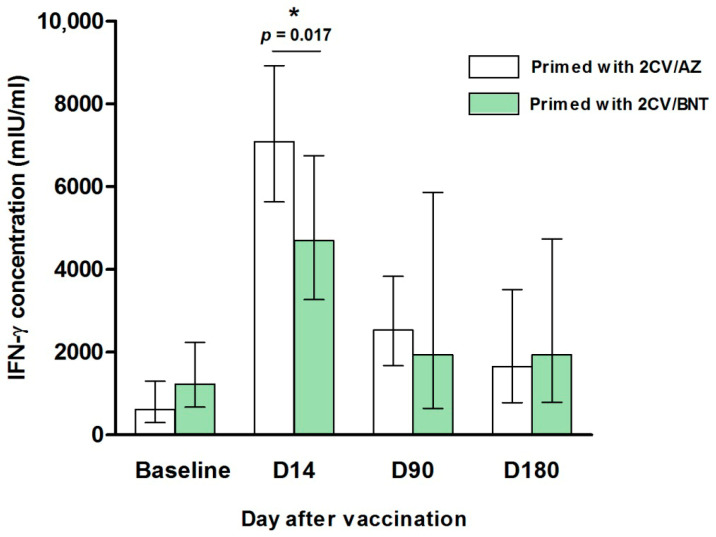
IFN-γ concentration before, and days 14 (D14), D90, and D180 after the fourth booster vaccination with mRNA-1273 in participants primed with either 2 doses of CoronaVac followed by AZ (2CoronaVac/AZ) or 2 doses of CoronaVac followed by BNT (2CoronaVac/BNT) (*n* = 10 per group). Bars represent the median, and error bars denote the interquartile range (IQR). Statistical differences between independent groups were assessed using the Mann–Whitney U test. * *p* < 0.05 compared with primed with 2CoronaVac/AZ group (white bar).

Figure 5 presents IFN-γ concentrations at baseline and from D14 to D180 following the mRNA-1273 booster. At baseline, 9 out of 10 participants in the 2CV/AZ-primed group and all 10 participants in the 2CV/BNT-primed group had positive IGRA results (defined as >200 mIU/mL). The median baseline IFN-γ concentration was 614.76 mIU/mL (IQR: 292.16–1293.58) in the 2CV/AZ group and 1224.65 mIU/mL (IQR: 670.58–2236.52) in the 2CV/BNT group.

At D14, 90, and 180 following the mRNA-1273 booster, all participants in both groups continued to show positive IGRA results. In the 2CV/AZ-primed group, the median IFN-γ concentrations were 7085.16 mIU/mL (IQR: 5628.95–8918.10) at D14, 2531.49 mIU/mL (IQR: 1671.50–3833.92) at D90, and 1652.31 mIU/mL (IQR: 777.99–3509.21) at D180. In the 2CV/BNT-primed group, the corresponding median IFN-γ concentrations at D14, D90, and D180 were 4693.55 mIU/mL (IQR: 3266.36–6744.33), 1932.00 mIU/mL (IQR: 637.58–5856.54), and 1930.58 mIU/mL (IQR: 787.09–4735.31), respectively. At D14, T cell responses were significantly higher in the 2CV/AZ-primed group compared to the 2CV/BNT-primed group (*p* = 0.017). However, by D90 and 180, T cell responses had declined in both groups and showed no significant difference between groups.

## 4. Discussion

The current study presents crucial scientific evidence regarding the immunogenicity and safety of a fourth dose (second booster) of the heterologous COVID-19 vaccine regimen using the mRNA-1273 vaccine in a cohort of Thai adults who had previously received two doses of the inactivated vaccine CoronaVac, followed by a third booster dose of either AZD1222 or BNT162b2. At the time of the study, data on this specific vaccination strategy in the Thai population were severely lacking, making the results of this study particularly significant for public health policy planning. This is especially true during a period when the world was facing the Omicron variant of the SARS-CoV-2 virus, which is highly transmissible and has a greater ability to evade immunity than previous variants [24]. The research findings confirm the ability of the mRNA-1273 vaccine to induce a powerful immune response and demonstrate an acceptable safety profile in a population that had already received multiple vaccine types. The details of the immune response, including both antibody and T-cell responses, as well as the correlation between laboratory results and the real-world epidemic situation at the time, are presented and analyzed in depth in the following sections.

This study showed that a fourth dose of mRNA-1273 significantly increased viral inhibition levels in both 2CV/AZ and 2CV/BNT groups within 14 days of vaccination. In the 2CV/AZ group, inhibition of the Delta variant rose from 51.93% to 99.88%, while in the 2CV/BNT group it increased from 71.68% to 99.79%. Similar trends were observed for anti-RBD IgG levels, which rose sharply in both groups at day 14. These findings are consistent with previous reports demonstrating that mRNA boosters effectively stimulate immunity in individuals primed with inactivated or viral vector vaccines [21].

For Omicron variants, the geometric mean of sVNT inhibition also increased at day 14 post-booster, with no significant difference between groups, indicating that mRNA-1273 can elicit robust immune responses against Omicron [25,26]. However, follow-up at 90 and 180 days revealed a progressive decline in inhibition in both groups, while responses against Delta remained consistently above 80%, consistent with earlier studies [27,28]. These findings are in line with earlier studies demonstrating that mRNA boosters elicit strong neutralization against Omicron, which may reduce gradual waning over time.

Long-term findings showed a clear difference in the durability of immunity against different viral variants. Inhibition of the Delta variant remained high for a sustained period of up to 180 days in both groups, a result of the original vaccine’s target on the spike protein of the ancestral virus, which is highly similar to the Delta variant [29]. In contrast, inhibition of the Omicron variant declined steadily and rapidly in both groups, particularly in the 2CV/AZ group. This rapid decrease in immunity reflects the biological property of the Omicron variant’s immune evasion, which involves extensive mutations in the spike protein, especially in the receptor-binding domain (RBD), the primary target for neutralizing antibodies [30]. Although the booster effectively increased the response to Omicron initially, this rapid decline in immune levels is a significant indication that immunity from the original vaccines may be insufficient to provide sustained protection against highly immune-evasive variants. This finding is consistent with reports from multiple studies that demonstrate a rapid decline in vaccine effectiveness against Omicron infection, even after receiving 3–4 doses of the vaccine [31].

These results highlight two key aspects. First, mRNA-1273 booster vaccination is highly effective in eliciting rapid and strong neutralizing antibody responses, even in individuals with weaker initial immunity. Second, although such responses remain durable against the Delta variant, waning immunity against Omicron raises concerns regarding long-term protection and suggests the need for updated booster formulations or additional doses to maintain cross-variant effectiveness. These findings reinforce the importance of booster campaigns using mRNA platforms and support the ongoing development of variant-adapted vaccines to address immune escape by emerging SARS-CoV-2 strains.

At baseline, the GMT of anti-RBD IgG was significantly lower in the 2CV/AZ-primed group compared with the 2CV/BNT group. By day 14 post-mRNA-1273 booster, however, IgG titers in the 2CV/AZ-primed group increased substantially and even surpassed those in the 2CV/BNT group, despite their lower baseline levels. This suggests that heterologous boosting with an mRNA vaccine following viral vector priming can elicit humoral responses that are not only comparable to, but in some cases greater than, those achieved with homologous mRNA-based regimens. Supporting evidence comes from the COV-BOOST trial in the United Kingdom, which demonstrated that mRNA boosters administered after viral vector priming induced antibody and T-cell responses comparable to or exceeding those from homologous mRNA regimens [18]. Similarly, Pozzetto et al. reported that heterologous vaccination with a viral vector followed by mRNA produced stronger humoral immunity and broader T-cell responses compared with homologous schedules [32]. Collectively, these findings underscore the value of heterologous prime–boost strategies, particularly the use of mRNA vaccines as boosters after viral vector priming, in eliciting potent and effective immune responses against SARS-CoV-2 [33].

Notably, in the Thai context, where inactivated vaccines were widely deployed as the primary series, heterologous booster strategies offer both immunological and programmatic advantages. Boosting with alternative platforms, such as mRNA or viral vector vaccines, enhances immune responses and provides important flexibility in settings with limited or variable vaccine supply. The ability to use different vaccine platforms interchangeably allows vaccination programs to proceed without delay, thereby supporting timely and efficient vaccine rollout. In this context, heterologous boosting represents not only an immunological approach but also a pragmatic strategy to optimize vaccine deployment while maintaining effective protection against COVID-19.

This study evaluated cell-mediated immune responses using an interferon-gamma release assay (IGRA) following an mRNA-1273 booster in individuals primed with either 2CV/AZ or 2CV/BNT. At baseline, nearly all participants demonstrated positive IGRA results, confirming that both heterologous priming regimens induced measurable T-cell immunity. Previous studies have shown that viral vector and mRNA COVID-19 vaccines generate a predominantly Th1 CD4+ T-cell response that persists for up to six months, similar to the kinetics observed after natural infection [34,35]. After the mRNA-1273 booster, IFN-γ concentrations rose markedly in both groups, underscoring the capacity of mRNA vaccines to enhance T-cell responses. Notably, at day 14 post-booster, participants primed with 2CV/AZ had significantly higher median IFN-γ concentrations than those primed with 2CV/BNT. This likely reflects the effect of viral vector priming, which has been reported to induce stronger T-cell responses than mRNA priming. The greater post-booster expansion in the 2CV/AZ group suggests that viral vector priming established a more durable pool of memory T cells that responded more vigorously upon re-stimulation with mRNA-1273 [34,36]. Consistent with this, prior studies have demonstrated that adenoviral vector priming elicits robust T-cell responses, whereas B-cell responses, including antibody titers, tend to be lower than those induced by mRNA priming [37].

In this study, local and systemic reactogenicity following a full-dose (100 µg) mRNA-1273 booster was commonly reported and was comparable between the 2CV/AZ- and 2CV/BNT-primed groups. Pain at the injection site was the most frequent local reaction, while systemic symptoms most often included myalgia, fatigue, headache, and fever. These findings align with previous reports describing the reactogenic profile of mRNA-1273, particularly at the higher 100 µg dosage [38]. Although the overall frequency of adverse events was high, most were mild to moderate in severity and self-limiting. Severe adverse reactions were uncommon, highlighting the acceptable safety profile of the mRNA-1273 booster in both heterologous priming groups. Furthermore, rare adverse events, including autoimmune reactions and thrombotic events, have been reported with certain COVID-19 vaccines; however, no such events were observed in our study. These results support an overall balance between immunogenicity and tolerability, indicating that while full-dose mRNA-1273 boosters may elicit greater short-term reactogenicity, they remain safe and well tolerated in the studied population.

The study found that a significant number of participants were excluded from the long-term analysis due to SARS-CoV-2 infection during the study period, which coincided with the Omicron outbreak in Thailand. This finding is consistent with the immune-evasive characteristics of Omicron, which have been associated with reduced protection against infection despite prior vaccination [10,12]. Although our immunogenicity data demonstrated robust antibody and T cell responses following the mRNA-1273 booster, the high rate of breakthrough infections during the Omicron wave suggests that vaccine-induced immunity was insufficient to provide complete protection against divergent variants. However, viral sequencing of breakthrough cases was not performed to confirm the infecting variant, and clinical severity was not evaluated. Thus, the relationship between variant type, immune responses, and clinical outcomes cannot be determined.

It is noteworthy that vaccine-induced immune responses are influenced by the degree of antigenic matching between the vaccine strains and circulating viral variants. Given the high specificity of vaccines, their effectiveness may be reduced when antigenic drift results in mismatches with emerging variants. Therefore, continuous monitoring of circulating strains and ongoing evaluation of vaccine performance remain essential to ensure optimal protection.

This study has limitations that should be acknowledged. First, the relatively small sample size, particularly in the cell-mediated immunity subgroup, limits the generalizability of the findings and restricts detailed subgroup analyses. In addition, the assessment was based on surrogate immune markers (sVNT and IGRA) rather than direct clinical endpoints such as vaccine effectiveness against infection or disease severity. Second, the exclusion of many participants due to breakthrough infections during the study period disrupted the continuity of the longitudinal data.

## 5. Conclusions

A heterologous fourth booster with mRNA-1273, administered after two doses of CoronaVac and a third dose of AZD1222 or BNT162b2, elicited strong humoral and T-cell responses with an acceptable safety profile. Neutralizing antibodies against Delta persisted for up to 180 days, whereas responses to Omicron declined more rapidly, particularly in individuals without prior mRNA priming. These findings highlight the importance of booster doses or variant-adapted vaccines to sustain protection against emerging SARS-CoV-2 variants.

## Figures and Tables

**Table 1:** Baseline characteristics of the study participants.

Baseline Characteristics	Total (*n* = 97)	Post 2CV/AZ (*n* = 47)	Post 2CV/BNT (*n* = 50)	*p *-Value
Age median y (IQR)	39 (31–44)	40 (31–45) )	37.5 (29–43)	0.267
Female, n (%)	79 (81.44)	44 (88.00)	35 (74.47)	0.118
BMI, median (IQR)	23.23 (20.20–27.48)	24.03 (20.57–28.37)	22.53 (20.06–25.69)	0.169
- < 25 kg/m^2^, *n* (%)	61 (64.21)	27 (55.10)	34 (73.91)	
- ≥ 25 kg/m^2^, *n* (%)	34 (35.79)	22 (44.90)	12 (26.09)	
Comorbidities, *n* (%)	25 (26.32)	15 (30.61)	10 (21.74)	0.360
- Allergic rhinitis	10 (10.53)	4 (8.16)	6 (13.04)	1.000
- Diabetes mellitus	5 (5.26)	3 (6.12)	2 (4.35)	1.000
- Obesity	4 (4.21)	4 (8.16)	0 (0.00)	0.118
- CVS/hypertension	3 (3.16)	2 (4.08)	1 (2.17)	1.000
- Thyroid	2 (2.06)	1 (2.00)	1 (2.13)	0.516
- Chronic lung disease	1 (1.05)	1 (2.04)	0 (0.00)	1.000
Interval between 3rd dose of AZD1222 or BNT162b2 to mRNA1273 booster (day); median (IQR)	164 (153–185)	184 (149–190)	162 (158–164)	0.005 **
sVNT to Delta variant (%inhibition), GM (95% CI)	60.71 (54.66–67.43)	51.93 (44.63–60.43)	71.68 (62.58–81.98)	<0.001 ***
sVNT to Omicron variant (%inhibition), GM (95% CI)	24.55 (19.46–30.95)	18.57 (12.45–27.69)	29.56 (22.35–39.10)	<0.001 ***
Anti-S-RBD IgG (BAU/mL), GM (95% CI)	804.89 (696.38–930.31)	640.70 (518.95–791.02)	1025.99 (857.18–1228.04)	0.003 **

AZ: AstraZeneca COVID-19 Vaccine; BMI: Body mass index; BNT: BNT162b2 vaccine; CI: Confidence interval; CV: CoronaVac vaccine; GM: Geometric mean; *n*: number of participants; IQR: Interquartile range; S-RBD: Spike receptor binding domain; sVNT: surrogate virus neutralization test. Statistical comparisons between groups were performed using the Wilcoxon rank-sum test (age, BMI, and interval time between the third and fourth dose), chi-square (χ^2^) test or Fisher’s exact test (sex, BMI, and comorbidities), and independent *t*-test (antibody titers). Represented significant ** *p* < 0.01, *** *p* < 0.001.

## Data Availability

All data supporting the findings of this study are included within the article.

## Supplementary Materials

The following supporting information can be downloaded at: https://www.mdpi.com/article/10.3390/vaccines14040348/s1, Table S1: Local and systemic reactogenicities within 7 days after the fourth booster dose of mRNA-1273 among individuals primed with two doses of CoronaVac followed by either AZD1222 (CV/AZ) or BNT162b2 (CV/BNT).
