# Supplementary material for: Comparison of Immune Responses and Safety Profiles Following a Fourth Heterologous Dose (Second Booster) with mRNA-1273 in Individuals Previously Vaccinated with Two Doses of CoronaVac and a Booster Dose of Either AZD1222 or BNT162b2
Source: Vaccines (Basel). 2026 Apr 15;14(4):348. doi: 10.3390/vaccines14040348 (PMC13119892; doi:10.3390/vaccines14040348)
Supplement: Supplementary file 1 [file vaccines-14-00348-s001.zip › vaccines-4229927-supplementary.pdf]

Table S1. Local and systemic reactogenicities within 7 days after the fourth booster dose of mRNA-1273 among individuals primed with two doses of CoronaVac followed by either AZD1222 (CV/AZ) or BNT162b2 (CV/BNT).

Side effect	Total N (%)	Post 2CV/ AZ (N = 47)	Post 2CV/ BNT (N =50)	*p*-value
Fever				
- No fever	62 (63.92)	34 (68.00)	28 (59.57)	
- Grade 1	20 (20.62)	9 (18.00)	11 (23.40)	0.663
- Grade 2	11 (11.34)	6 (12.00)	5 (10.64)	
- Grade 3	4 (4.12)	1 (2.00)	3 (6.38)	
Pain at injected site				
- No pain	4 (4.12)	3 (6.00)	1 (2.13)	
- Grade 1	29 (29.90)	14 (28.00)	15 (31.91)	0.068
- Grade 2	55 (56.70)	25 (50.00)	30 (63.83)	
- Grade 3	9 (9.28)	8 (16.00)	1 (2.13)	
Swelling at injected site				
- No	89 (91.75)	45 (90.00)	44 (93.62)	
- Grade 1	6 (6.19)	4 (8.00)	2 (4.26)	0.838
- Grade 2	2 (2.06)	1 (2.00)	1 (2.13)	
- Grade 3	0 (0.00)	0 (0.00)	0 (0.00)	
Redness at injected site				
- No	92 (94.85)	46 (92.00)	46 (97.87)	
- Grade 1	1 (1.03)	1 (2.00)	0 (0.00)	0.618
- Grade 2	4 (4.12)	3 (6.00)	1 (2.13)	
- Grade 3	0 (0.00)	0 (0.00)	0 (0.00)	
Headache				
- No	32 (32.99)	17 (34.00)	15 (31.91)	
- Grade 1	24 (24.74)	14 (28.00)	10 (21.28)	0.830
- Grade 2	37 (38.14)	17 (34.00)	20 (42.55)	
- Grade 3	4 (4.12)	2 (4.00)	2 (4.26)	
Fatigue				
- No	25 (25.77)	14 (28.00)	11 (23.40)	
- Grade 1	30 (30.93)	17 (34.00)	13 (27.66)	0.769
- Grade 2	37 (38.14)	17 (34.00)	20 (42.55)	
- Grade 3	5 (5.15)	2 (4.00)	3 (6.38)	
Myalgia				
- No	20 (20.62)	15 (30.00)	5 (10.64)	0.119
- Grade 1	25 (25.77)	12 (24.00)	13 (29.55)	
- Grade 2	45 (46.39)	20 (40.00)	25 (53.19)	
- Grade 3	7 (7.22)	3 (6.00)	4 (8.51)	
Joint pain				
- No	79 (81.44)	45 (90.00)	34 (72.34)	
- Grade 1	10 (10.31)	3 (6.00)	7 (14.89)	0.098
- Grade 2	6 (6.19)	1 (2.00)	5 (10.64)	
- Grade 3	2 (2.06)	1 (2.00)	1 (2.13)	
Vomiting				
- No	92 (94.85)	47 (94.00)	45 (95.74)	
- Grade 1	5 (5.15)	3 (6.00)	2 (4.26)	1.000
- Grade 2	0 (0.00)	0 (0.00)	0 (0.00)	
- Grade 3	0 (0.00)	0 (0.00)	0 (0.00)	
Diarrhea				
- No	85 (87.63)	42 (84.00)	43 (91.49)	
- Grade 1	11 (11.34)	7 (14.00)	4 (8.51)	0.526
- Grade 2	1 (1.03)	1 (2.00)	0 (.00)	
- Grade 3	0 (0.00)	0 (0.00)	0 (0.00)	

N = number of subjects. Adverse events grading according to U.S. Department of Health and Human Services F, CBER. Guidance for Industry Toxicity Grading Scale for Healthy Adult and Adolescent Volunteers Enrolled in Preventive Vaccine Clinical Trials September 2007 [Available from: https://www.fda.gov/media/73679/download.Accessed date 30 November, 2021].
